# Charles William Sargent, PhD, AHIP, FMLA, 1925–2020

**DOI:** 10.5195/jmla.2020.1010

**Published:** 2020-10-01

**Authors:** Teresa L. Knott

**Affiliations:** 1 tlknott@vcu.edu, Interim Dean and University Librarian, VCU Libraries, Virginia Commonwealth University, Richmond, VA

## Abstract

Charles William Sargent, PhD, AHIP, FMLA, director emeritus of the Texas Tech University Health Sciences Center Library of the Health Sciences and 1981–1982 president and member of the 1972–1975 Board of Directors of the Medical Library Association (MLA), died on January 8, 2020, in San Antonio, Texas. Sargent's career in librarianship included positions at research, special, and health sciences libraries, as well as an appointment as a library school faculty member. He was known for his mentoring, interest in clinical librarianship, and belief in the power of organizations.

Charles William Sargent, PhD, AHIP, FMLA, director emeritus of the Texas Tech University Health Sciences Center Library of the Health Sciences and 1981–1982 president of the Medical Library Association (MLA), died on January 8, 2020, in San Antonio, Texas, after a brief illness. Sargent was born on December 18, 1925, in Shelburn, Indiana, and spent his childhood in Flint, Michigan. He received bachelor's and master's degrees in history from Michigan State University (MSU) before matriculating to the University of Michigan (UM), where he received his master's degree in library science in 1953. In 1964, he earned a doctoral degree in economic history from the University of New Mexico [1].

Sargent's career in librarianship included positions at research, special, and health sciences libraries, as well as an appointment as a library school faculty member. He worked at large research libraries including MSU, UM, and the University of Kansas–Lawrence before relocating to New Mexico to work at the Sandia Corporation Library as the chief of cataloging. Sargent's journey as a medical librarian began when he was recruited to join the Lovelace Clinic Department of Aerospace Medicine as an information scientist. From there, he moved onto a more traditional track, serving as the deputy director at the University of New Mexico's medical library. In this time period, he consulted for Donald A. B. Lindberg, the future director of the National Library of Medicine. Lindberg recruited Sargent to lead the Information Science Department at the University of Missouri–Columbia.

**Figure:**
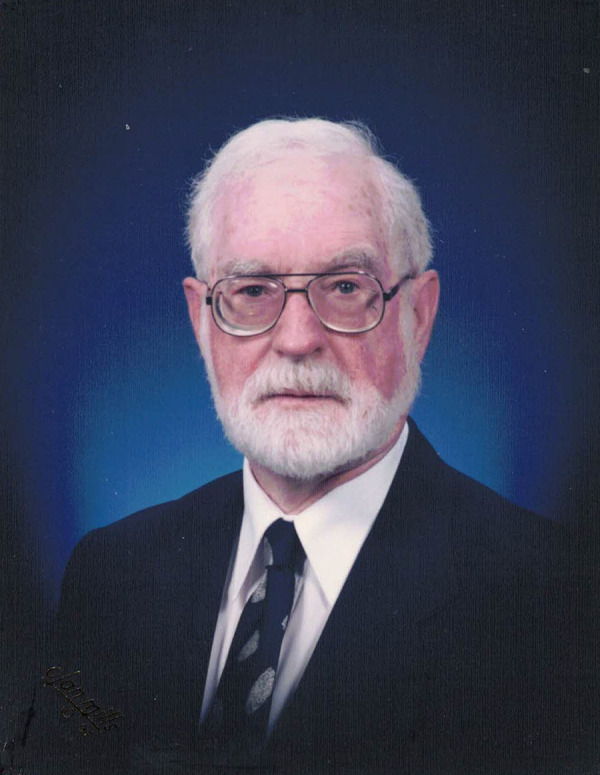


As the Information Science Department chair, Sargent successfully competed for a National Library of Medicine grant to train medical librarians from 1968–1972. His commitment to training medical librarians was reflected in his sustained interest in mentoring health sciences librarians [2].

As Deborah Ward, interim vice provost for libraries and director of Health Sciences Library at the University of Missouri, explained:

He was a mentor to an entire group of young librarians, many of whom went on to prestigious positions of their own. It's to his credit that he encouraged us all to be forward-looking leaders with a sense of accountability. [3]

In 1972, he was appointed the inaugural library director for the newly established Texas Tech University Health Sciences Center (TTUHSC) and chair of the Texas Tech University School of Medicine Department of Health Communications. He retained these positions until his retirement in 1990 [2]. He strove to keep the Library of the Health Sciences (TTUHSC Library) at the forefront of libraries by automating library processes, implementing services such as integration of medical librarians into clinical care teams, and expanding the concept of the library beyond the bounds of the physical space.

In today's culture, Sargent would likely be regarded as a technology geek. He proudly deployed the first IBM personal computers in Lubbock to TTUHSC Library staff. TTUHSC was the first multicampus library system to implement the Georgetown Library Information System to automate acquisitions, circulation, and serials. Naomi C. Broering, AHIP, FMLA, retired director of Georgetown University's Dahlgren Library, explained that Sargent:

actively inspired many library directors to adopt the concept of integrated library information systems to help users search for literature…He began a momentum of library leadership at universities and hospitals to automate and integrate their systems. This changed the future direction of libraries as influential leaders in the information revolution of the 1980s–'90s and onward. [4]

Having worked with Gertrude Lamb at the University of Missouri, he adopted her passion for clinical medical librarianship and initiated a successful program with the TTUHSC School of Medicine that included family medicine, internal medicine, and orthopedic surgery. In his MLA oral history, Sargent shared:

My concept of the Library with the walls removed is that it gives you an opportunity to do a great many things, specifically outside of the traditional things that librarians have done. The image that a library has is of the books and the journals on rows and rows of shelves. Data now is taking a variety of forms, digital as well as print form…This is an opportunity to store, disseminate, coordinate and be the broker for all kinds of information. [5]

Ursula K. Scott, founding associate director, TTUHSC Library at Odessa, Texas, and university librarian emeritus, Uniformed Services University of the Health Sciences Library, noted:

I appreciated that he was open to new ideas and technologies. He encouraged his employees to develop and implement new concepts. He was good at taking on new opportunities for librarian engagement afforded by the administration. It was a creative time and he let his staff explore and do new, creative things. [6]

As a member of the 1972–1975 MLA Board of Directors and the 1981–1982 MLA president, Sargent led the association through significant organizational upheaval. The 1980–1981 MLA president suffered serious health issues during the year, which necessitated Sargent taking on additional responsibilities as president-elect [7]. Sargent's term on the board coincided with high inflation in the United States, and he referenced the financial challenges ahead for MLA in his inaugural address, remarking that MLA members had rejected a dues increase in 1980. The board implemented stringent economic measures for the association [8].

In his presidential address, Sargent expounded on additional challenges that MLA faced. He had to hire a new MLA executive director and a director of education, which helped mitigate the financial constraints in 1981–1982. Sargent hired Raymond Palmer, who served as MLA executive director from February 1982–December 1991. The salary savings from vacant positions kept the organization on the positive side of the financial ledger. Other challenges that Sargent addressed as president included the resignations of the editors for the *Bulletin of the Medical Library Association* and the *MLA News*. Both positions were filled by the conclusion of Sargent's term.

With these challenges met, Sargent set the organization on a positive path forward. Section Council and Chapter Council were formed in 1980. Although the MLA Bylaws did not detail board responsibilities for the council chairs, Sargent appointed the council chairs to board committees to share their expertise and to actively participate on the board, establishing a model for engagement that continues through the present [9].

Additionally, during his presidential term, the board adopted a strategic planning process with an ambitious timeline. Strategic priorities that were established in 1982 resonate with today's environment: providing further educational opportunities, improving librarian status through educating employers and advocating for better compensation, making annual meetings more meaningful, strengthening relationships with the National Library of Medicine, and promoting basic research in medical librarianship. Sargent appointed the first MLA committee on research in concert with these strategic goals. A new special interest group dedicated to research was also founded [10].

A theme that runs through Sargent's life and career was a deeply held belief in the power of organizations. He was a great organizer. If he belonged to an organization, he was an active participant and leader. If an organization did not exist that he thought was needed, he would collaborate to create it. Sargent was involved in creating the South Central Regional Group of MLA (SCRG) and chaired the committee that developed bylaws for the nascent organization in 1973 [11]. Later, he chaired the SCRG and was recognized with the Distinguished Service Award in 1988 [2]. In fall 1982, he was a founding member and first chair of the South Central Academic Medical Libraries Consortium (SCAMeL). A key SCAMeL initiative was creating and maintaining a union list of serials for member organizations, an activity contracted to the TTUHSC Library [12].

In retirement, he was engaged in his retirement community's governance and initiated social and travel groups in the community. After relocating to San Antonio, Texas, Sargent served on the board of directors for the University of Texas Health Science Center at San Antonio P. I. Nixon Medical Historical Library [2]. Ward summed it up with:

He had a sense of purpose that drove him to bring people together in creating organizations, from SCAMeL and the Georgetown Users Group at the regional and national levels, to the local Toastmasters and Lubbock Area Libraries Association. [3]

As a leader, Sargent was goal oriented, was innovative, and advocated for preparing to meet future challenges. In his presidential address, he challenged MLA members by saying:

I hope that you, as well as all the members, take up the challenge of greatness that can be MLA, an association whose members must be considered in all future matters pertaining to the delivery of health information. [10]

Family and community were vitally important to Sargent. While working as an assistant circulation librarian at MSU, Sargent met his future wife, Nanette Reed. Reed and Sargent married in November 1950 and were married for sixty-six years. Together, they shared a deep faith and a passion for travel, opera, reading, and libraries. They traveled the world in keeping with what Sargent said was his family motto, “See the world you are in before you leave it.” Sargent is survived by his son and daughter-in-law, Edward and Julia; three grandchildren; and two great-grandchildren [13].
